# Supplementary material for: Maternal age and body mass index and risk of labor dystocia after spontaneous labor onset among nulliparous women: A clinical prediction model
Source: PLoS One. 2024 Sep 6;19(9):e0308018. doi: 10.1371/journal.pone.0308018 (PMC11379172; doi:10.1371/journal.pone.0308018)
Supplement: S1 Table — (PDF) [file pone.0308018.s001.pdf]

S1 Table Definition and description of predictors and outcome

Predictor	Definition	Unit/Levels	Primary source	Secondary source	Expected effect
Maternal age	Age in years at time of birth	continuous	MBR		Higher risk
Pre-pregnancy BMI	BMI prior to pregnancy in kg/m²	continuous	CPC	MBR	Higher risk
Height	Height in cms	Cut-off ≤160[a]	CPC		Higher risk
Gestational age	Gestational age in weeks + days on day of birth	five groups:<br>37+0-37+6<br>38+0-38+6<br>39+0-39+6<br>40+0-40+6<br>≥41+0	MBR		Higher risk
Physical activity	Question: Do you at present engage in physical activity? If yes, indicate type (*prespecified*) and hours weekly.	three groups:<br>0 hours<br><3.5 hours<br>≥3.5 hours[b]	CPC		Lower risk
Medical condition, somatic	Defined as a positive answer to any of the following prespecified main disease categories: hypertension, lung disease, type 1 diabetes, type 2 diabetes, endocrinologic disease, rheumatological disease, epilepsy, cardiac disease, migraine, other disease	yes/no	CPC		Higher risk
Medical condition, psychiatric	Defined as a positive answer to the prespecified disease category: mental illness	yes/no	CPC		Higher risk
WHO-5 Well-being Index Score	Self-reported psychological well-being measured by the five item World Health Organization's Well-being Index. Scale from 0 (lowest possible well-being) to 100 (highest possible well-being)	Cut-off ≤50[c]	CPC		Lower risk
Fertility treatment	Question: Have you in this present pregnancy been treated for infertility with e.g. hormones, IVF, ICSI, insemination or operation?	yes/no	CPC		Higher risk
**Outcome**	**Definition**	**Medical code**	**Source[d]**		
Labour dystocia	Oxytocin augmentation	SKS code: BKHD3, BKHD31	MBR (2014-2018)		
	Dystocia diagnosis	ICD-10 code: O62* abnormalities of forces of labour	MBR (2019-2020)		

Abbreviations: Copenhagen Pregnancy Cohort, CPC; The Danish Medical Birth Registry, MBR; Sundhedsvæsenets Klassifikation system (translates to The Danish Healthcare Classification System), SKS

[a]Cut-off based on study in Kjærgaard et al.[39]

[b]The Danish National Health Authorities recommend 30 minutes of daily physical activity of moderate intensity throughout pregnancy. The group reporting ≥3.5 hours adheres to this recommendation.[41]

[c]Using a cut-off score of ≤50 the WHO-5 has high sensitivity and specificity for depression and is used the screening of this.[42]

[d]Coding is based on the Danish Healthcare Classification System including the International Classification of Disease (version 10) (ICD-10).[43]
