# Supplementary material for: Maternal age and body mass index and risk of labor dystocia after spontaneous labor onset among nulliparous women: A clinical prediction model
Source: PLoS One. 2024 Sep 6;19(9):e0308018. doi: 10.1371/journal.pone.0308018 (PMC11379172; doi:10.1371/journal.pone.0308018)
Supplement: S2 Table — (PDF) [file pone.0308018.s002.pdf]

S2 Table Selected model input with associated coefficients and standard error

Predictors	Coefficient	Standard error
Intercept	-1.86	0.52
BMI[a]	0.02	0.02
BMI	0.03	0.02
Age	0.05	0.01
Age	0.02	0.02
Physical activity		
<3.5	-0.08	0.07
≥3.5	-0.12	0.05
GA		
37+0-37+6	0.03	0.10
38+0-38+6	-0.11	0.08
39+0-39+6	-0.08	0.06
≥41+0	0.47	0.06
Height >160	-0.37	0.07
Medical condition		
Psychiatric	0.25	0.12
Somatic	0.28	0.08
Both	0.27	0.18
WHO-5 ≤50	0.00	0.06
Fertility treatment	-0.05	0.07

Abbreviations: BMI, Body mass index; GA, gestational age

[a]BMI and age appear twice due to the application of splines.
