# Supplementary material for: Maternal age and body mass index and risk of labor dystocia after spontaneous labor onset among nulliparous women: A clinical prediction model
Source: PLoS One. 2024 Sep 6;19(9):e0308018. doi: 10.1371/journal.pone.0308018 (PMC11379172; doi:10.1371/journal.pone.0308018)
Supplement: S3 Table — (PDF) [file pone.0308018.s003.pdf]

**Table S3** Predicted risk for labour dystocia for selected combinations of age and BMI*

| **Age** years | **BMI** kg/m² | **GA** weeks+days | **Physical activity** hours weekly | **Height** cm | **Medical condition** | **Fertility treatment** | **WHO-5 score** score | **Predicted risk** |
|---|---|---|---|---|---|---|---|---|
| 20 | 20 | 40+0-40+6 | ≥3.5 | >160 | None | No | >50 | 25.0% |
| 21 | 20 | 40+0-40+6 | ≥3.5 | >160 | None | No | >50 | 25.9% |
| 22 | 20 | 40+0-40+6 | ≥3.5 | >160 | None | No | >50 | 26.8% |
| 23 | 20 | 40+0-40+6 | ≥3.5 | >160 | None | No | >50 | 27.7% |
| 24 | 20 | 40+0-40+6 | ≥3.5 | >160 | None | No | >50 | 28.6% |
| 25 | 20 | 40+0-40+6 | ≥3.5 | >160 | None | No | >50 | 29.5% |
| 26 | 20 | 40+0-40+6 | ≥3.5 | >160 | None | No | >50 | 30.5% |
| 27 | 20 | 40+0-40+6 | ≥3.5 | >160 | None | No | >50 | 31.5% |
| 28 | 20 | 40+0-40+6 | ≥3.5 | >160 | None | No | >50 | 32.5% |
| 29 | 20 | 40+0-40+6 | ≥3.5 | >160 | None | No | >50 | 33.5% |
| 30 | 20 | 40+0-40+6 | ≥3.5 | >160 | None | No | >50 | 34.7% |
| 31 | 20 | 40+0-40+6 | ≥3.5 | >160 | None | No | >50 | 36.0% |
| 32 | 20 | 40+0-40+6 | ≥3.5 | >160 | None | No | >50 | 37.4% |
| 33 | 20 | 40+0-40+6 | ≥3.5 | >160 | None | No | >50 | 38.9% |
| 34 | 20 | 40+0-40+6 | ≥3.5 | >160 | None | No | >50 | 40.4% |
| 35 | 20 | 40+0-40+6 | ≥3.5 | >160 | None | No | >50 | 42.1% |
| 36 | 20 | 40+0-40+6 | ≥3.5 | >160 | None | No | >50 | 43.7% |
| 37 | 20 | 40+0-40+6 | ≥3.5 | >160 | None | No | >50 | 45.4% |
| 38 | 20 | 40+0-40+6 | ≥3.5 | >160 | None | No | >50 | 47.1% |
| 39 | 20 | 40+0-40+6 | ≥3.5 | >160 | None | No | >50 | 48.8% |
| 40 | 20 | 40+0-40+6 | ≥3.5 | >160 | None | No | >50 | 50.4% |
| 20 | 21 | 40+0-40+6 | ≥3.5 | >160 | None | No | >50 | 25.4% |
| 21 | 21 | 40+0-40+6 | ≥3.5 | >160 | None | No | >50 | 26.3% |
| 22 | 21 | 40+0-40+6 | ≥3.5 | >160 | None | No | >50 | 27.2% |
| 23 | 21 | 40+0-40+6 | ≥3.5 | >160 | None | No | >50 | 28.1% |
| 24 | 21 | 40+0-40+6 | ≥3.5 | >160 | None | No | >50 | 29.0% |

| | | | | | | | | |
|---|---|---|---|---|---|---|---|---|
| 25 | 21 | 40+0-40+6 | ≥3.5 | >160 | None | No | >50 | 30.0% |
| 26 | 21 | 40+0-40+6 | ≥3.5 | >160 | None | No | >50 | 30.9% |
| 27 | 21 | 40+0-40+6 | ≥3.5 | >160 | None | No | >50 | 31.9% |
| 28 | 21 | 40+0-40+6 | ≥3.5 | >160 | None | No | >50 | 32.9% |
| 29 | 21 | 40+0-40+6 | ≥3.5 | >160 | None | No | >50 | 34.0% |
| 30 | 21 | 40+0-40+6 | ≥3.5 | >160 | None | No | >50 | 35.2% |
| 31 | 21 | 40+0-40+6 | ≥3.5 | >160 | None | No | >50 | 36.5% |
| 32 | 21 | 40+0-40+6 | ≥3.5 | >160 | None | No | >50 | 37.9% |
| 33 | 21 | 40+0-40+6 | ≥3.5 | >160 | None | No | >50 | 39.4% |
| 34 | 21 | 40+0-40+6 | ≥3.5 | >160 | None | No | >50 | 41.0% |
| 35 | 21 | 40+0-40+6 | ≥3.5 | >160 | None | No | >50 | 42.6% |
| 36 | 21 | 40+0-40+6 | ≥3.5 | >160 | None | No | >50 | 44.2% |
| 37 | 21 | 40+0-40+6 | ≥3.5 | >160 | None | No | >50 | 45.9% |
| 38 | 21 | 40+0-40+6 | ≥3.5 | >160 | None | No | >50 | 47.6% |
| 39 | 21 | 40+0-40+6 | ≥3.5 | >160 | None | No | >50 | 49.3% |
| 40 | 21 | 40+0-40+6 | ≥3.5 | >160 | None | No | >50 | 51.0% |
| 20 | 22 | 40+0-40+6 | ≥3.5 | >160 | None | No | >50 | 26.0% |
| 21 | 22 | 40+0-40+6 | ≥3.5 | >160 | None | No | >50 | 26.9% |
| 22 | 22 | 40+0-40+6 | ≥3.5 | >160 | None | No | >50 | 27.8% |
| 23 | 22 | 40+0-40+6 | ≥3.5 | >160 | None | No | >50 | 28.7% |
| 24 | 22 | 40+0-40+6 | ≥3.5 | >160 | None | No | >50 | 29.6% |
| 25 | 22 | 40+0-40+6 | ≥3.5 | >160 | None | No | >50 | 30.6% |
| 26 | 22 | 40+0-40+6 | ≥3.5 | >160 | None | No | >50 | 31.6% |
| 27 | 22 | 40+0-40+6 | ≥3.5 | >160 | None | No | >50 | 32.6% |
| 28 | 22 | 40+0-40+6 | ≥3.5 | >160 | None | No | >50 | 33.6% |
| 29 | 22 | 40+0-40+6 | ≥3.5 | >160 | None | No | >50 | 34.7% |
| 30 | 22 | 40+0-40+6 | ≥3.5 | >160 | None | No | >50 | 35.9% |
| 31 | 22 | 40+0-40+6 | ≥3.5 | >160 | None | No | >50 | 37.2% |
| 32 | 22 | 40+0-40+6 | ≥3.5 | >160 | None | No | >50 | 38.6% |
| 33 | 22 | 40+0-40+6 | ≥3.5 | >160 | None | No | >50 | 40.1% |
| 34 | 22 | 40+0-40+6 | ≥3.5 | >160 | None | No | >50 | 41.7% |
| 35 | 22 | 40+0-40+6 | ≥3.5 | >160 | None | No | >50 | 43.3% |

| | | | | | | | | |
|---|---|---|---|---|---|---|---|---|
| 36 | 22 | 40+0-40+6 | ≥3.5 | >160 | None | No | >50 | 45.0% |
| 37 | 22 | 40+0-40+6 | ≥3.5 | >160 | None | No | >50 | 46.7% |
| 38 | 22 | 40+0-40+6 | ≥3.5 | >160 | None | No | >50 | 48.3% |
| 39 | 22 | 40+0-40+6 | ≥3.5 | >160 | None | No | >50 | 50.0% |
| 40 | 22 | 40+0-40+6 | ≥3.5 | >160 | None | No | >50 | 51.7% |
| 20 | 23 | 40+0-40+6 | ≥3.5 | >160 | None | No | >50 | 26.7% |
| 21 | 23 | 40+0-40+6 | ≥3.5 | >160 | None | No | >50 | 27.6% |
| 22 | 23 | 40+0-40+6 | ≥3.5 | >160 | None | No | >50 | 28.6% |
| 23 | 23 | 40+0-40+6 | ≥3.5 | >160 | None | No | >50 | 29.5% |
| 24 | 23 | 40+0-40+6 | ≥3.5 | >160 | None | No | >50 | 30.5% |
| 25 | 23 | 40+0-40+6 | ≥3.5 | >160 | None | No | >50 | 31.4% |
| 26 | 23 | 40+0-40+6 | ≥3.5 | >160 | None | No | >50 | 32.4% |
| 27 | 23 | 40+0-40+6 | ≥3.5 | >160 | None | No | >50 | 33.4% |
| 28 | 23 | 40+0-40+6 | ≥3.5 | >160 | None | No | >50 | 34.5% |
| 29 | 23 | 40+0-40+6 | ≥3.5 | >160 | None | No | >50 | 35.6% |
| 30 | 23 | 40+0-40+6 | ≥3.5 | >160 | None | No | >50 | 36.8% |
| 31 | 23 | 40+0-40+6 | ≥3.5 | >160 | None | No | >50 | 38.1% |
| 32 | 23 | 40+0-40+6 | ≥3.5 | >160 | None | No | >50 | 39.5% |
| 33 | 23 | 40+0-40+6 | ≥3.5 | >160 | None | No | >50 | 41.0% |
| 34 | 23 | 40+0-40+6 | ≥3.5 | >160 | None | No | >50 | 42.6% |
| 35 | 23 | 40+0-40+6 | ≥3.5 | >160 | None | No | >50 | 44.3% |
| 36 | 23 | 40+0-40+6 | ≥3.5 | >160 | None | No | >50 | 45.9% |
| 37 | 23 | 40+0-40+6 | ≥3.5 | >160 | None | No | >50 | 47.6% |
| 38 | 23 | 40+0-40+6 | ≥3.5 | >160 | None | No | >50 | 49.3% |
| 39 | 23 | 40+0-40+6 | ≥3.5 | >160 | None | No | >50 | 51.0% |
| 40 | 23 | 40+0-40+6 | ≥3.5 | >160 | None | No | >50 | 52.7% |
| 20 | 24 | 40+0-40+6 | ≥3.5 | >160 | None | No | >50 | 27.7% |
| 21 | 24 | 40+0-40+6 | ≥3.5 | >160 | None | No | >50 | 28.6% |
| 22 | 24 | 40+0-40+6 | ≥3.5 | >160 | None | No | >50 | 29.5% |
| 23 | 24 | 40+0-40+6 | ≥3.5 | >160 | None | No | >50 | 30.5% |
| 24 | 24 | 40+0-40+6 | ≥3.5 | >160 | None | No | >50 | 31.4% |
| 25 | 24 | 40+0-40+6 | ≥3.5 | >160 | None | No | >50 | 32.4% |

| | | | | | | | | |
|---|---|---|---|---|---|---|---|---|
| 26 | 24 | 40+0-40+6 | ≥3.5 | >160 | None | No | >50 | 33.4% |
| 27 | 24 | 40+0-40+6 | ≥3.5 | >160 | None | No | >50 | 34.4% |
| 28 | 24 | 40+0-40+6 | ≥3.5 | >160 | None | No | >50 | 35.5% |
| 29 | 24 | 40+0-40+6 | ≥3.5 | >160 | None | No | >50 | 36.6% |
| 30 | 24 | 40+0-40+6 | ≥3.5 | >160 | None | No | >50 | 37.8% |
| 31 | 24 | 40+0-40+6 | ≥3.5 | >160 | None | No | >50 | 39.2% |
| 32 | 24 | 40+0-40+6 | ≥3.5 | >160 | None | No | >50 | 40.6% |
| 33 | 24 | 40+0-40+6 | ≥3.5 | >160 | None | No | >50 | 42.1% |
| 34 | 24 | 40+0-40+6 | ≥3.5 | >160 | None | No | >50 | 43.7% |
| 35 | 24 | 40+0-40+6 | ≥3.5 | >160 | None | No | >50 | 45.4% |

*The table presents a selection of unique combinations. A full overview is available by contacting the corresponding author or via access to the data repository Open Science Framework: Available from: https://osf.io/kq7um/

Abbreviations: BMI, body mass index; GA, gestational age

All predictors, other than BMI and age, are retained.
