# Supplementary figures and images for: Maternal age and body mass index and risk of labor dystocia after spontaneous labor onset among nulliparous women: A clinical prediction model
Source: PLoS One. 2024 Sep 6;19(9):e0308018. doi: 10.1371/journal.pone.0308018 (PMC11379172; doi:10.1371/journal.pone.0308018)

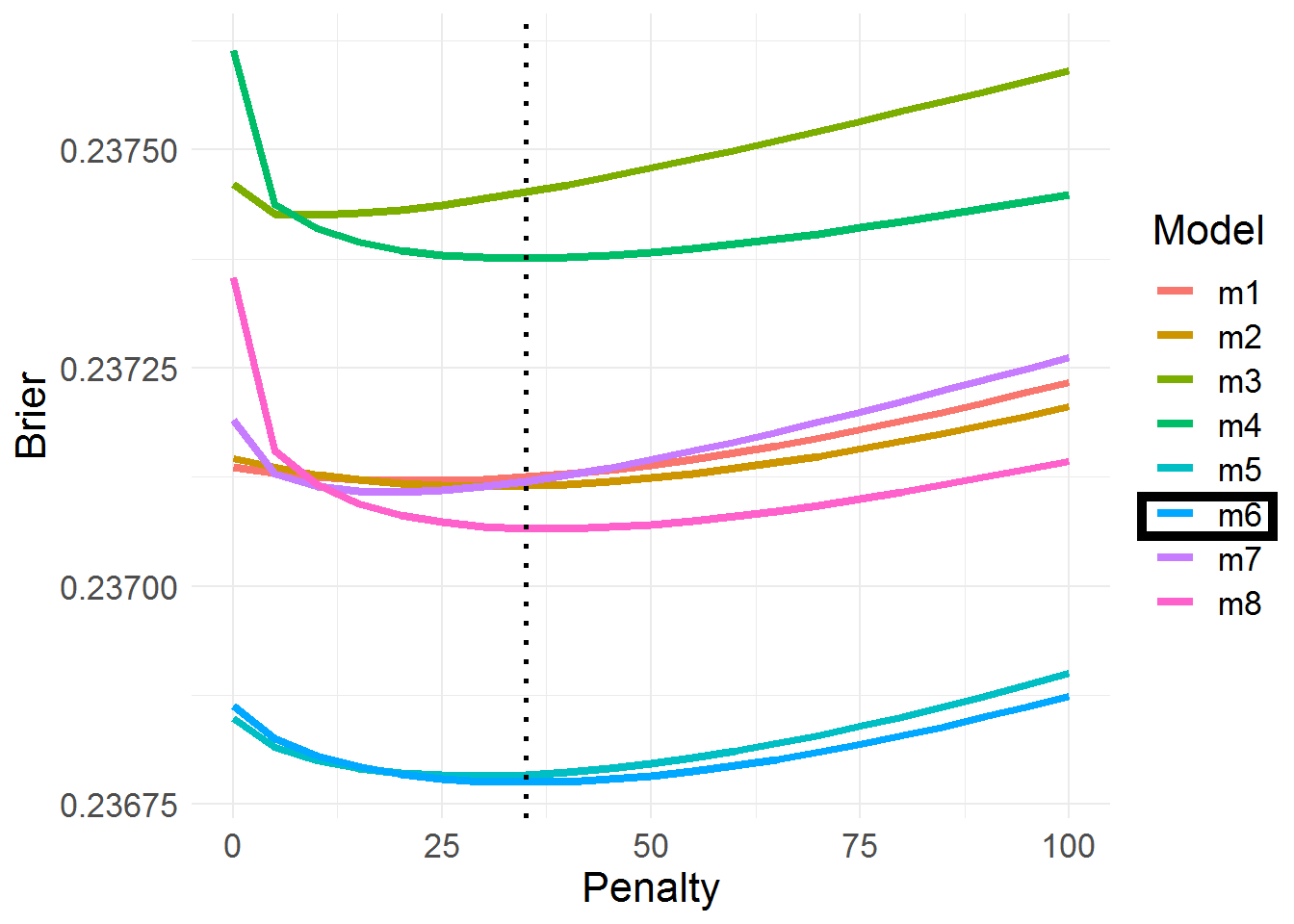

Supplement: S1 Fig — Abbreviation: m, model. Model six was the final selected model for which model performance was tested in validation dataset. See Table 1 for specifications for all eight candidate models. (TIF) [file pone.0308018.s004.tif]

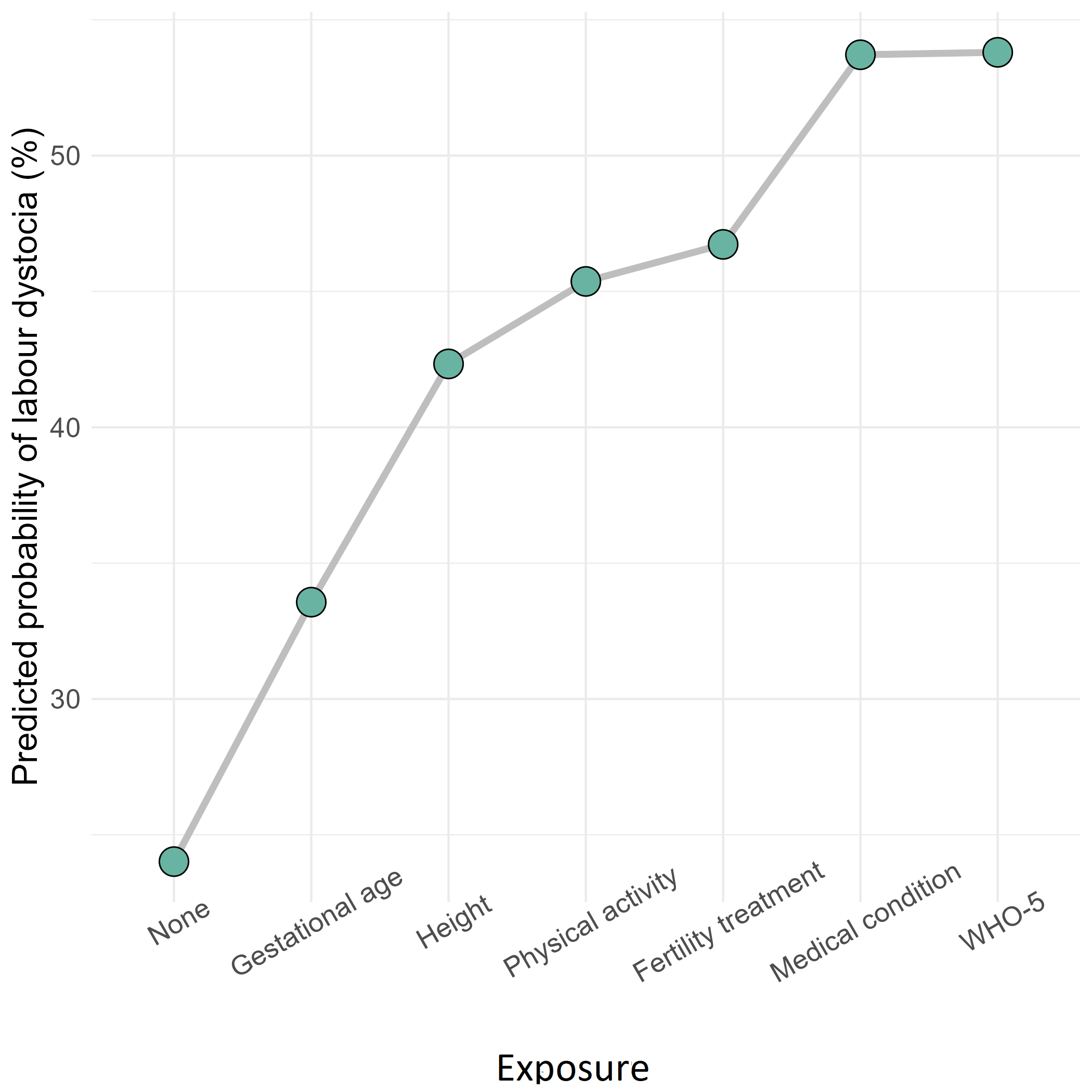

Supplement: S2 Fig — The figure shows the effect of ‘switching on’ exposures starting from ‘None’ which is the low-risk scenario defined in Table 3. Only categorical candidate predictors are incorporated in the figure. The order is as follows: Gestational age from 40+0–40+6 to ≥41+0; Height from >160cm to ≤160cm; Physical activity from ≥3.5 hours weekly to no physical activity; Fertility treatment from yes to no; Medical condition from none to somatic; WHO-5 score from >50 to ≤50. (TIF) [file pone.0308018.s005.tif]

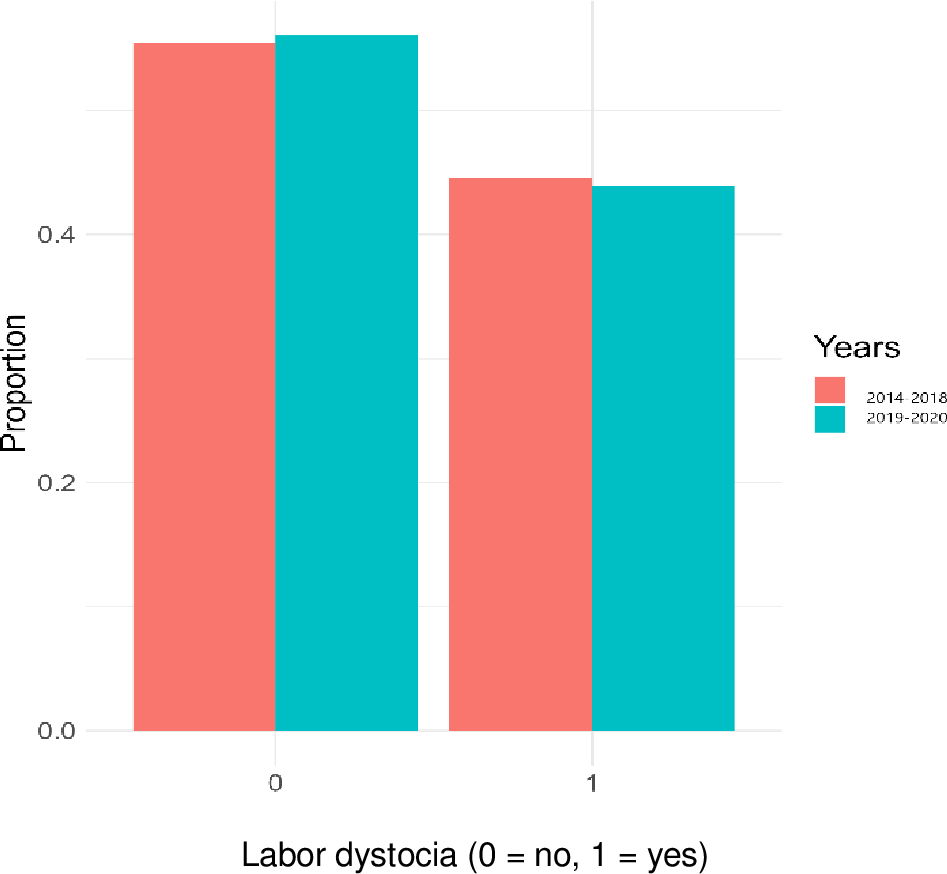

Supplement: S3 Fig — In the years 2014–2018, the outcome is defined by oxytocin augmentation, while from year 2019 the outcome is defined by ICD-10 diagnosis codes. (TIF) [file pone.0308018.s006.tif]
